# Evacuation Optimization Strategy for Large-Scale Public Building Considering Plane Partition and Multi-Floor Layout

**DOI:** 10.3389/fpubh.2022.847399

**Published:** 2022-02-21

**Authors:** Han Fang, Wei Lv, He Cheng, Xiaolian Li, Bingjie Yu, Zhongwei Shen

**Affiliations:** ^1^School of Architecture, Southwest Jiaotong University, Chengdu, China; ^2^School of Safety Science and Emergency Management, Wuhan University of Technology, Wuhan, China; ^3^Fujian Police College, Fuzhou, China

**Keywords:** evacuation, public safety, public place, optimization strategy, architectural programming

## Abstract

Large-scale public buildings (e.g., stadiums and comprehensive hospitals) in modern cities provide places for various social activities. However, all of these public places encounter the scenario of large passenger flow and crowd gathering, which is highly likely to induce serious safety problems, such as stampedes. Previous studies have shown that efficient evacuation is an important way to ensure the safety of dense crowds in public places. This study aims to explore the optimization methods to improve the evacuation efficiency of public buildings. Two strategies considering plane partition and multi-floor layout are proposed for plane evacuation and vertical evacuation, respectively. Simulation scenarios and models of large stadiums and high-rise hospitals are established to verify the strategies. The results show that plane partition could effectively shorten the total evacuation time, which is due to the optimization of the initial exit choice of individuals and the avoidance of regional congestion in some evacuation channels or exits. Multi-floor layout optimization is an effective management method to arrange the different features of different floors, which could improve the evacuation efficiency for the whole multi-floor building. This study is helpful for building designers and managers to improve the building space layout design and the daily safety management mode.

## Introduction

Public places provide convenience for public life. But emergencies and great safety hazards in public places may cause serious injuries (1, 2). A stampede in a stadium in Manila killed 73 people. The fire in the Grenfell tower in London in 2017 killed 79 people. These accidents indicated that when large-space public places and high-rise buildings face emergencies, pedestrians need to be evacuated to a safe area in the shortest possible time, which can easily cause squeezing and trampling accidents. Therefore, effective evacuation guidance and evacuation layout are important means to improve the efficiency of pedestrian evacuation and ensure the safety of pedestrians.

The evacuation behavior of crowds is a hot issue in evacuation research. Many scholars have carried out a lot of research on pedestrian evacuation in public places. From the movement characteristics of evacuees, the evacuation forms in public places can be divided into two types: plane evacuation and vertical evacuation. In the study of plane evacuation, related scholars have carried out research on pedestrian evacuation through experiments, models, optimization strategies, and other methods. The experiment of Cuesta et al. (3) showed that large groups have higher evacuation efficiency than small groups and individuals. Zou et al. (4) analyzed pedestrian evacuation through the cellular automata model and pointed out that pedestrian speeds can alter the evacuation efficiency to a certain extent. Using the FGCAE model, Fang et al. (5) simulated the evacuation process of Airbus A380 to evaluate the evacuation efficiency of different exit locations. Wu et al. (6) pointed out that proper physical separation measures can effectively reduce the congestion at the exit in the early evacuation period and found that the symmetrical distribution of the exit is beneficial to improve the evacuation efficiency. Liu et al. (7) considered the distance between pedestrians and hazards to study the movement behavior of pedestrians and pointed out that the closer the pedestrian to the danger hazard, the higher the speed, and the reasonable arrangement of obstacles in space can effectively reduce the evacuation time. What is more, research has found that the effect of the interior room door size becomes weaker as the building becomes larger, while the size of the main exit consistently plays a strong role in controlling the evacuation time (8). In the study of evacuation strategy, Kurdi et al. (9) introduced a balanced evacuation algorithm to ensure that pedestrians can safely evacuate through multiple exits, which can optimize the limitations of traditional strategies and help reduce congestion around exits. Lu et al. (10) used a stochastic utility model to model the occupant path selection. The simulation showed that the optimized guidance strategy could reduce the overall risk and evacuation time compared with the guided evacuation that does not consider the response of the evacuees. Wang et al. (11) considered the bounded rational route choice behavior of pedestrians during the multi-exit evacuation and examined the influence of congestion sensitivity and conservative level on evacuation efficiency. When the conservative level is low, pedestrians are more inclined to change the route, which can significantly reduce the evacuation time. Guo and Zhang (12, 13) proposed a simulation-based subway station evacuation evaluation and optimization method combining random forest (RF) and non-dominated sorting genetic algorithm III (NSGA-III). The research applied to subway evacuation pointed out that it can be very effective. It is suitable for use in risk assessment and evacuation optimization of subway stations.

In terms of vertical evacuation research, Huo et al. (14) conducted two experiments on the stairs of high-rise buildings: phased evacuation and total evacuation. The study pointed out that the movement speed of pedestrians going down the stairs will decrease due to the influx of people on the current floor. Ding et al. (15) pointed out that the evacuation from low-level to high-level priority can achieve a faster evacuation speed in most evacuation processes, but the total evacuation time increases. Ma et al. consider the influence of different stair usage ratios of evacuees on high floors on evacuation efficiency and points out a proper ratio of the building occupants is transported to the ground level by fast elevators while others are evacuated by stairs, the evacuation process can reach an optimized state (16, 17). Fang's research (18) demonstrated that the downward velocity is determined by three aspects: merging behavior in the entrance buffer of the stairwell, the strength of participants, and visibility in the stairwell. In its evacuation experiments on super high-rise buildings, it is proved that compared with males, females are more sensitive to changes in vertical evacuation height (19), and the travel distance will affect descent speed (20). Model studies on evacuation efficiency show that human behaviors, including the arrival rate at the floor exit the decision-making on refuge floors, play an important role in affecting the evacuation efficiency (21); the evacuation time of 1,050 pedestrians under the situation of fatigue is almost twice of that under the situation of constant speed (22); evasive behavior between heterogeneous pedestrians (23) and physical isolation facilities (24) can reduce evacuation time.

These studies show that, in plane evacuation, considering group behavior and herd effect, the balanced use of exits and avoiding stampede behavior are the key issues of evacuation research (25, 26). In the vertical evacuation scene, evacuees move through elevators and stairs. A reasonable evacuation strategy can reduce the evacuation time of high-rise buildings. In this article, consider stadiums and high-rise hospitals as typical public places, which have different personnel composition and completely different evacuation characteristics, and establish models respectively, corresponding to their different evacuation optimization needs, and propose corresponding evacuation strategies.

## Models

In the study of plane evacuation, the stadium has the characteristics of both crowded pedestrians and concentrated evacuation needs. During the evacuation, problems such as crowded people and unbalanced evacuation requirements of various exits are more prominent. This section takes a large stadium as an example to carry out research. In the vertical evacuation modeling, considering the characteristics of the hospital with both dense and complex personnel composition, the modeling is carried out by taking a high-rise hospital as an example. Hospitals and stadiums are typical comprehensive public places with the characteristics of complex personnel composition, complex evacuation environment, and diverse path choices. By studying the models of these two scenarios, we can take into account problems facing most public place evacuation and propose corresponding strategies to improve evacuation efficiency.

### Stadium Model

The organizational form of crowd evacuation in stadiums can be divided into macro and micro levels. The forms of micro-evacuation organization include the design of corridors and seats and the arrangement of safety exits. Different designs and layouts can directly affect the overall efficiency of crowd evacuation. From the macro perspective, the form of evacuation organization refers to the use of distribution and guidance for the overall evacuees to organize the evacuation. According to the size of the stadium and the requirements for space utilization, the organizational situation will be different. The classification of the macro evacuation organization of the stadium is shown in Table 1. Different stadiums generally place the evacuation exits at the first or second-floor stands according to different building structures and functions. The stadium scene studied in this paper adopts the composite evacuation organization form.

**Table 1:** Macroscopic evacuation organization form of the stadium.

**Forms**	**Schematic**	**Features**
Evacuate from the upper floor	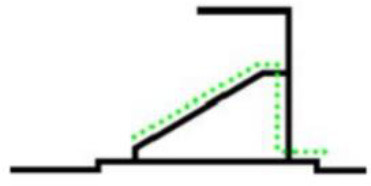	(1) The space utilization rate of the auditorium is very high. (2) Great physical consumption for evacuees during evacuation.
Evacuate from the middle	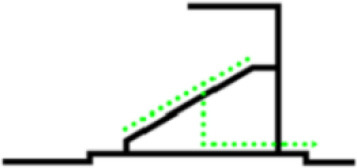	(1) The average evacuation distance is short. (2) Because of the space occupied by the evacuation exit, the high-rise stands need to be raised to a certain height.
Evacuate from the ground floor	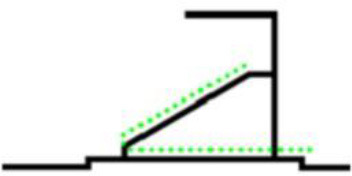	(1) There is no need to set stairs for high-rise evacuees. (2) Confluence phenomenon exists in the evacuation process.
Evacuate from both sides	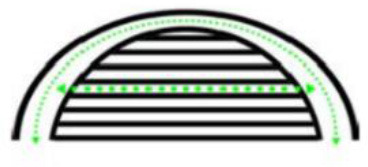	Ramps, compared with stairs, can reduce physical consumption during evacuation.
Composite evacuation	\	A composite evacuation form is a combination of two or more of the above forms.

**Figure 1:**
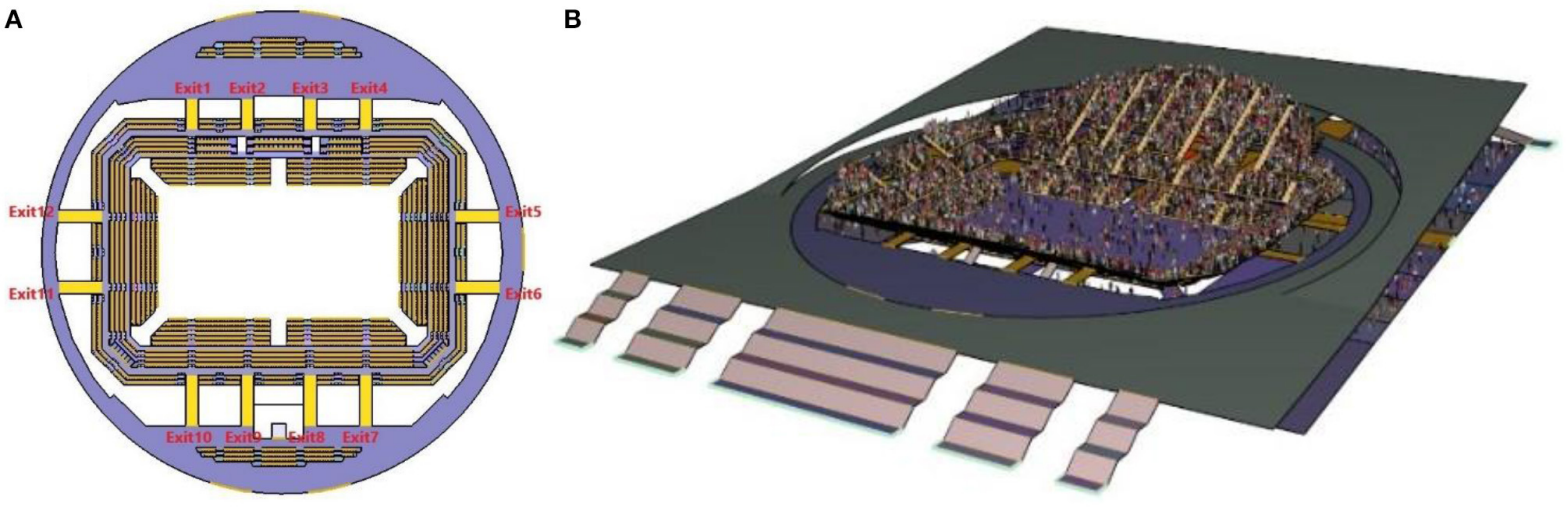
Stadium evacuation scene, **(A)** Top view **(B)** Three-dimensional view.

The stadium model diagram is shown in Figure 1. The floor area of the building is 18,000 m^2^, and the whole is divided into upper and lower floors. The first floor of the stadium is the sports area and work area, and the second floor is the audience seats. As the research object of this article, the second-floor grandstand can accommodate 4,043 seats and a total of 12 safety exits. The pathfinder evacuation simulation model of the stadium is established based on the DWG drawing file of the building. The first exit from the north left is numbered in clockwise order. The schematic diagram is shown in Figure 1A, and the width data of each safety exit is shown in Table 2. The pedestrian shoulder width parameter is set to 0.40 m, and the speed is set to 1.20 m/s.

**Table 2:** Width of each exit.

**Number of exits**	**1**	**2**	**3**	**4**	**5**	**6**	**7**	**8**	**9**	**10**	**11**	**12**
Width/m	2.20	2.11	2.15	2.06	2.02	2.14	2.05	2.19	2.01	2.12	2.03	2.20

### High-Rise Hospital Model

**Figure 2:**
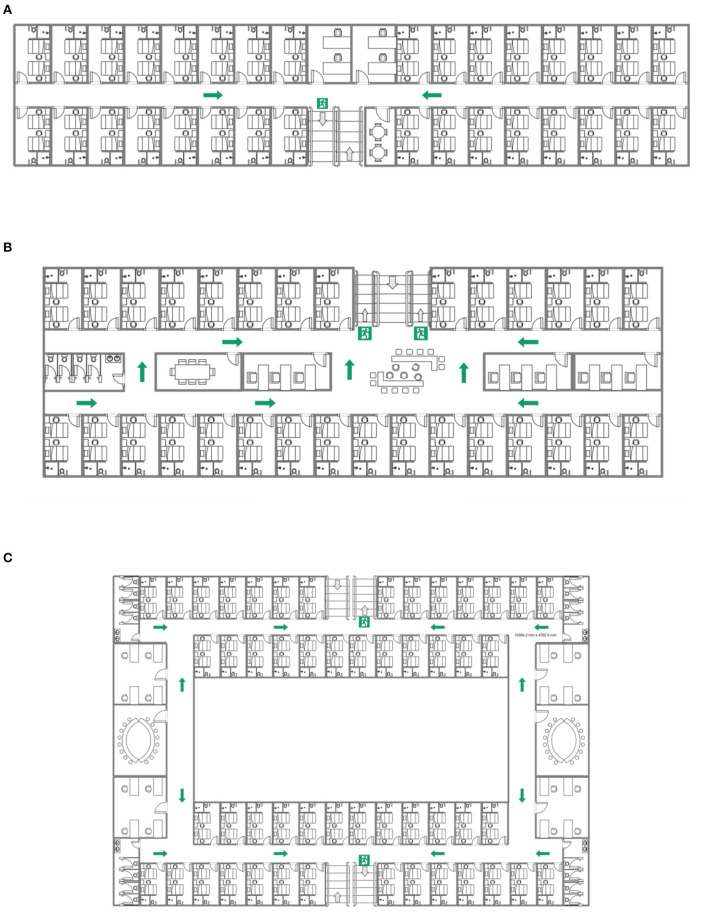
Different forms of plane space organization in the hospital. **(A)** Single corridor form. **(B)** Double corridor form. **(C)** Circular corridor form.

The plane space organization forms of high-rise hospitals mainly include single corridor form, double corridor form and circular corridor form (as shown in Figure 2).

In the form of a single corridor, a single corridor separates the ward and the medical auxiliary area to the north and south sides, and both the ward and the medical auxiliary area have sufficient lighting and ventilation. It is easy to identify the direction inside the building, which is conducive to evacuation. The disadvantage is that the length of the building from east to west is too long, which leads to the increase of the length of the evacuation path.

The double corridor form means that the three rows of rooms are separated by double corridors, the wards are arranged in the north and south directions, and the medical auxiliary area is located in the middle of the two corridors. Compared with the single corridor form, the building length is shortened, and the medical auxiliary area is located at the core position, which facilitates the flexible and effective access of nursing staff to each ward, and shortens the route length. However, its disadvantages lie in the lack of good sunshine conditions in the northward area, poor lighting and ventilation in the medical auxiliary area, requiring a lot of mechanical ventilation and artificial lighting, and high operating costs.

The circular corridor form is different from the traditional building form. The ward area is located in the north-south direction, and the medical auxiliary area is arranged in the east-west direction. Each ward has good lighting and ventilation. The atrium solves the problem of lighting and ventilation in the medical auxiliary area, but it is difficult to identify the direction inside the building, which can easily lead to getting lost.

Among the above three forms, the single corridor form is the most common, and the plane space organization form of the high-rise hospital in this paper is the single corridor.

**Figure 3:**
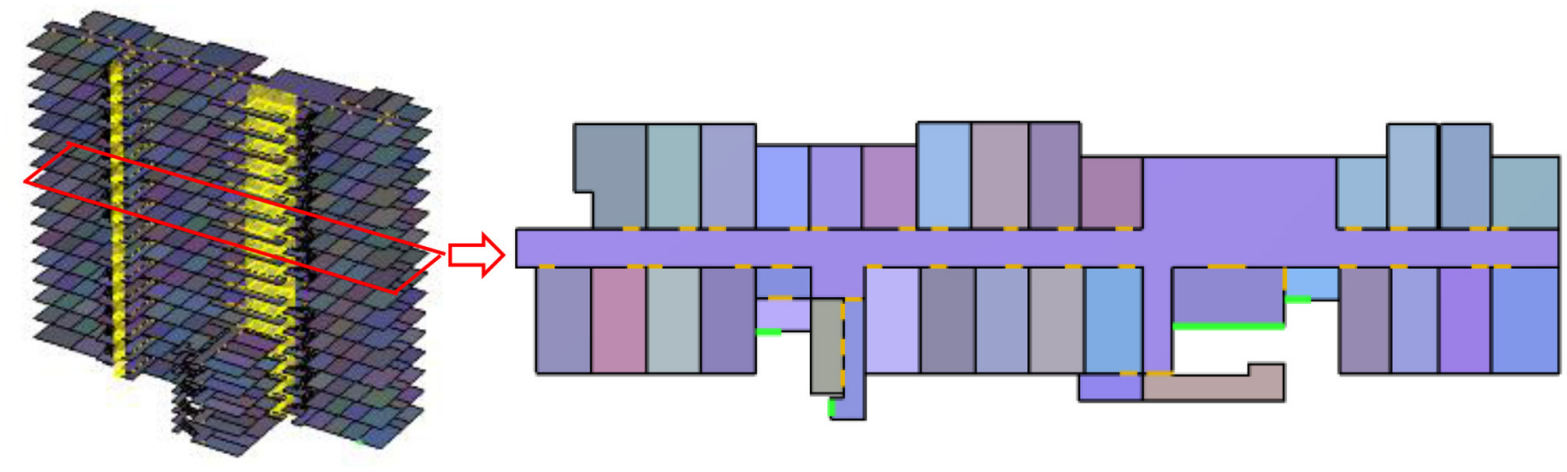
High-rise hospital scene.

The hospital scene established in this article has 16 floors. The first floor is the lobby, warehouse, pharmacy and various medical laboratories, and the second to the sixteenth floors are the wards of various departments. Among them, there are four exits on the first floor, and three evacuation stairs, four elevators are connected to the upper floors. Figure 3 is a top view of the hospital and a schematic diagram of the three-dimensional model.

**Figure 4:**
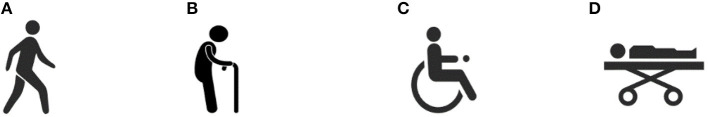
Schematic diagram of the types of evacuees. **(A)** Normal person. **(B)** Mild disabled person. **(C)** Severe disabled person. **(D)** Complete disabled person.

The hospital is a place where multiple types of pedestrians coexist. According to their exercise capacity, they can be divided into four types: normal person, mildly disabled person, severely disabled person, and completely disabled person, as shown in Figure 4. Among them, the normal person consists of patients with normal evacuation ability and medical staff. During the evacuation, medical staff will assist patients with limited evacuation ability to evacuate. Considering the individual differences of the people, the relevant data of various types of evacuees are shown in Table 3. Among them, the severely and complete disabled persons are unable to walk and need assistance from others. When a normal person assists the disabled to evacuate, the speed will be attenuated compared to the evacuation alone, which is the product of the normal evacuation speed and the reduction factor. The reduction factor is the deceleration ratio of pedestrians assisting patients in evacuation relative to normal walking. This article is set according to the default value of Pathfinder software.

**Table 3:** Parameters of various evacuees.

**Types**	**Plane projection width/mm**	**Velocity/(m/s)**	**Reduction factor**
Normal person	357~450	0.5~1.4	/
Mildly disabled person	357~441	0.3~1.1	/
Severely disabled person	760	/	0.7
Complete disabled person	900	/	0.4

## Optimization Strategy and Results

In this section, according to the plane evacuation and vertical evacuation models, corresponding optimized evacuation strategies are proposed, and the optimization results are analyzed.

### Plane Partition Evacuation

#### Optimization Strategy

In the evacuation scene of a large stadium, the position of the evacuees in the stadium is relatively fixed. When setting up the evacuation area, according to the location of the personnel inside the stadium, the number and width of the evacuation exits of the stadium, the layout of the seats and passages, etc., follow the principle that the time for the personnel to the evacuation exit is as short as possible. Divide people who use the same exit to the same area. In this scenario, there are a total of 2,935 seats in the north-south grandstand area, corresponding to eight evacuation exits, and a total of 1,108 seats in the east-west grandstand area, corresponding to four evacuation exits, so the evacuation exit capacity cannot match the corresponding number of seats. During the evacuation process, uneven use of exits often occurs, resulting in an increase in evacuation time. Theoretically, in the evacuation strategy of multi-exit buildings, the ideal evacuation effect can be achieved by balancing the evacuation efficiency of the exits and making the evacuation end time of each exit to be the same. Based on this, this paper proposes an optimized strategy method to divert the flow of people from high-utilization exits to adjacent low-utilization exits, balance the final evacuation time of each exit, and achieve the research goal of the final evacuation time of each exit of the scene being uniform.

In the initial simulation state, because of the long-distance difference between the various exits in the stadium, the evacuees choose the nearest evacuation exit to escape, so the path length from each pedestrian to each exit is calculated, and the shortest exit of the path is set as the initial choice of pedestrians.


(1) $$Ec_{n\;=\;}index(\min\left(D_{1}^{n,}D_{2}^{n}\;\ldots \;,D_{i}^{n}\right))(i=1,2,\ldots m)$$

Where *EC*_*n*_ indicates the exit number chosen by the *n*-th pedestrian; *D*${}_{i}^{n}$ indicates the path distance of the *n*-th pedestrian from the *i-*th exit, there are *m* exits in total; *index* indicates that the corresponding exit number is extracted according to the value of the path distance between the pedestrian and the exit.

According to the choice of pedestrian exits, calculate the average evacuation time and the optimal value of the ideal evacuation time. Starting from Exit 1, the pedestrians who have not been evacuated within the average evacuation time will be diverted to the adjacent exits in a clockwise sequence. If exit *i* completes the evacuation later than exit *i* + 1, the guidance process is shown in equation (2):


(2) $$\begin{array}{r} ECG_{i\to i+1}^{n}=\;RANK_{P\left(i\to i+1\right)\;}\left(\min D_{i+1}\right) \end{array}$$

Where *EGC*${}_{i\to i+1}^{n}$ indicates that the nth person who originally chose exit *i* was changed to the number of the person who chose exit *i* + *1*; min*D*_*i*+1_ indicates the minimum distance between pedestrians who originally chose exit *i* and exit *i* + 1;*RANK*_*P(i→ i+1)*_ indicates that the minimum distance is sorted, and the person with the top _*P(i→ i+1)*_ is selected. The average evacuation time of all evacuation exits is shown in Equation (3):


(3) $$\bar{T_{e}}=\;\sum_{i=1}^{m}T_{i,e}$$

*T*_*i, e*_ indicates the last evacuation time of exit *i*;$\bar{T}$ indicates the average of the last evacuation time of all evacuation exits. The average value of the average flow rate of adjacent exits is shown in Equation (4):


(4) $$\begin{array}{r} \bar{f}=\left(f_{i\;}+f_{i+1}\right)/2 \end{array}$$

*f*_*i*_ indicates the average flow rate of exit *i*; $\bar{f}$ represents the average value of the average flow rate of two adjacent exits; The standard deviation of the final evacuation time is given by formula (5):


(5) $$\begin{array}{r} P\left(i\to i+1\right)=\;\bar{f}\;\left(\bar{T_{e}}\;-T_{i+1,e}\right)/f_{i}\left(T_{i,e}-T_{i,s}\right) \end{array}$$

In the formula, *T*_*i, s*_ represents the evacuation time of exit *i*.

The optimization strategy starts with the guidance between Exit 1 and Exit 2 and ends with the guidance between Exit 12 and Exit 1 as a simulation iteration. Calculate the results of each simulation and calculate the standard deviation of the time required to complete the evacuation of all exits until the standard deviation does not change significantly at the end of the optimization.

#### Optimization Result

**Figure 5:**
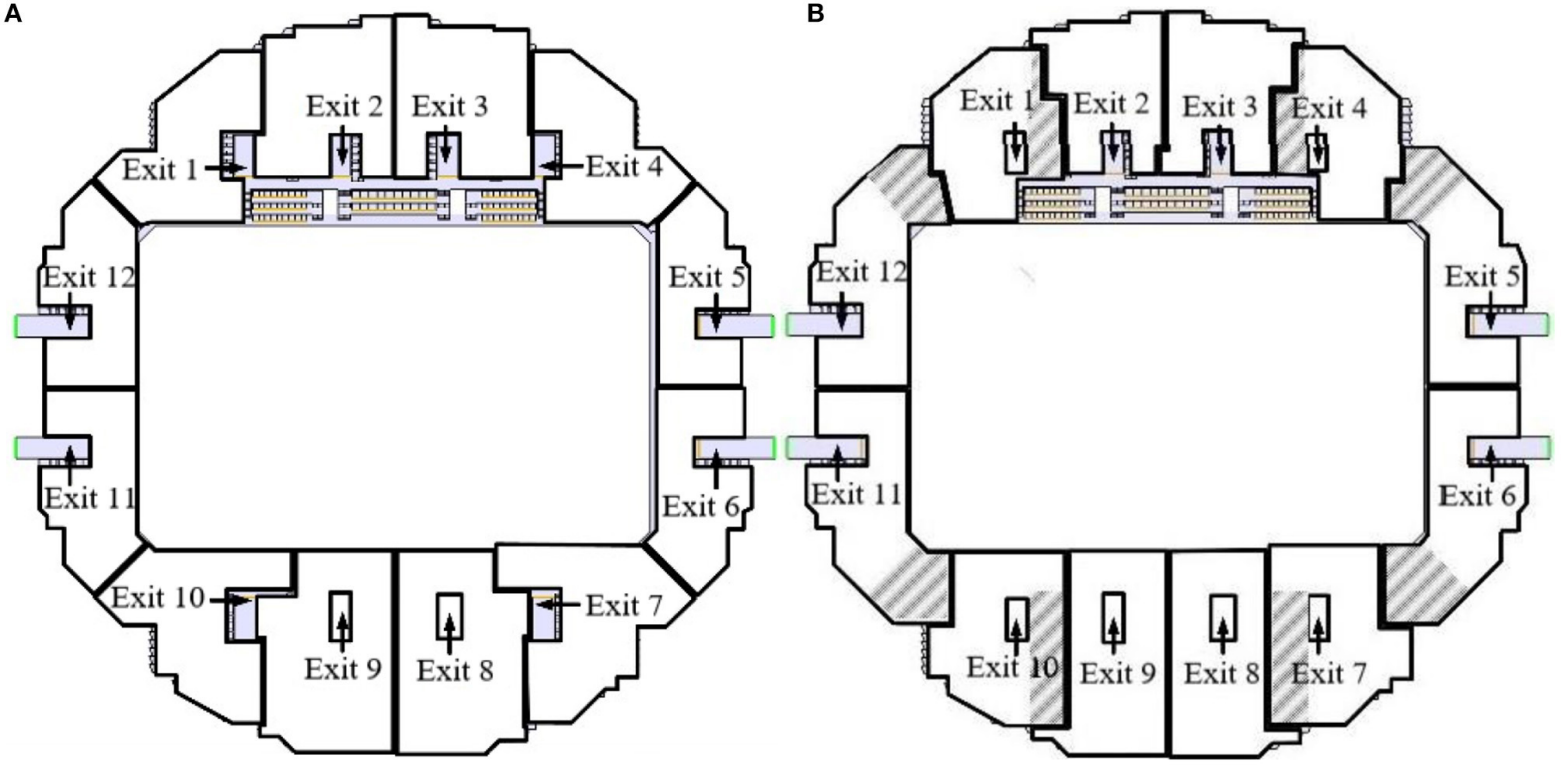
Exit location selection area partition, **(A)** Before optimization, **(B)** After optimization.

Calculate the shortest distance of each pedestrian relative to the 12 exits, and obtain the evacuation exit number selected by each pedestrian according to formula (1) to perform the initial evacuation zone in the stadium. The initial pedestrian exit selection of the stadium is shown in Figure 5. The stadium is a multi-exit evacuation scene. In the initial simulation evacuation scene of Pathfinder, any exit is selected as the evacuation exit under default conditions for all evacuation behaviors of people. If a person is to be evacuated only through designated exits, the exit selection in “Exit” needs to be changed from “Any” to “Choose.”

Before optimization, the evacuation situation of each exit is shown in Table 3. It can be seen from Table 3 that the exits began to evacuate within 5.3-7.3 s after the evacuation started. Exit 5 completed the evacuation at the earliest time at 92.4 s. The last pedestrian passed through exit 9 and reached the safe area to complete the complete evacuation at 179.2 s. The last use time difference between exit 9 and exit 5 is 86.8 s. During this time period, exit 9 is still in use, but exit 5 is idle, the flow rate of some exits is slower, and the congestion is more serious.

According to the pedestrian evacuation optimization strategy, after eight simulation iterations, the standard deviation of the final evacuation time of all exits is 2.99 s, and the standard deviation of the simulation iteration no longer changes significantly. The pedestrian exit selection of the stadium is shown in Figure 5B. It can be seen from the figure that the shaded area is the change of the area before and after optimization. Comparing (Figures 5A,B), the evacuation areas of exits 5, 6, 11, and 12 have been significantly expanded, and the exits have been fully utilized; the evacuation area of exit 4 is divided into exit 5, and some pedestrians at exit 3 choose to exit 4. After optimization, the evacuation situation of each exit is shown in Table 4. The complete evacuation time of the scene is 160.3 s, and the evacuation time difference between the earliest and the latest evacuation exit is 8.9 s. The evacuation capacity of each evacuation exit has been improved.

**Table 4:** Usage of each evacuation exit.

**Exit number**	**Before optimization**			**After optimization**		
	**Finally used/s**	**Total people/pers**	**Flow rate/(ped/m/s)**	**Finally used/s**	**Total people/pers**	**Flow rate/(ped/m/s)**
1	158.4	267	0.81	158.2	360	1.07
2	178.8	405	1.12	154.8	211	0.68
3	173.2	405	1.13	152.1	221	0.71
4	159.5	342	1.08	153.1	351	1.15
5	92.4	248	1.42	160.3	400	1.29
6	103.9	251	1.21	151.4	358	1.16
7	158.9	318	1.02	157	390	1.29
8	173.6	470	1.29	160.3	305	0.95
9	179.2	471	1.36	157.9	272	0.89
10	162.8	319	0.98	158	435	1.31
11	104	248	1.26	159.8	367	1.09
12	104.3	299	1.39	157.5	373	1.20

**Figure 6:**
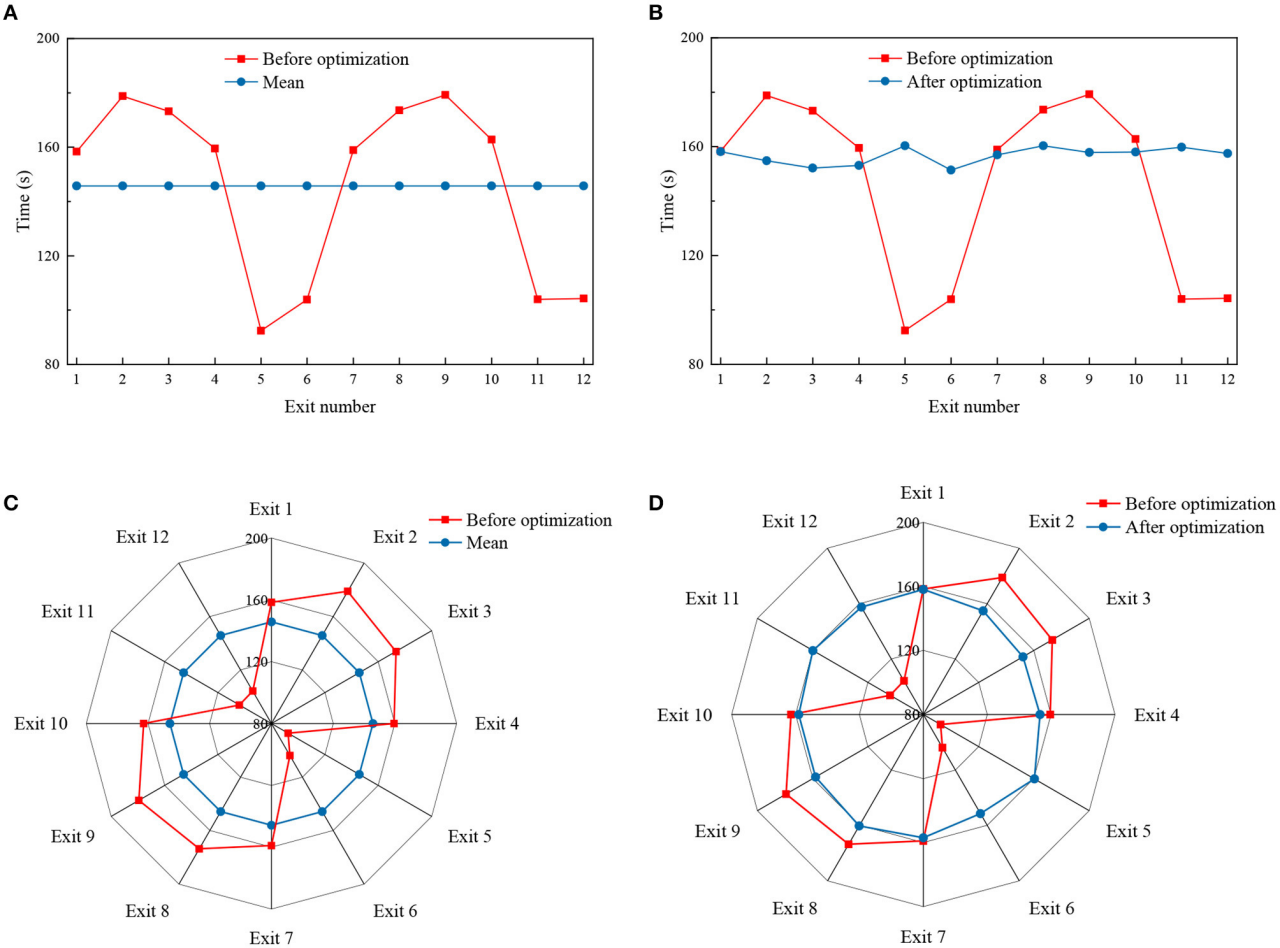
Comparison of evacuation time before and after optimization.

Table 4, Figure 6 show the changes in the evacuation exits and the overall evacuation completion time before and after optimization. It can be seen that the evacuation time before optimization was 179.2 s, and the standard deviation of evacuation time at each exit was 32.42 s; the evacuation time after optimization 160.3 s, the standard deviation of the evacuation time of each exit was 2.99 s, and the evacuation time was reduced by 10.55% after optimization. After the optimization of exits 2, 3, 8, and 9 near the wave crest, the evacuation time has decreased to a certain extent, and the evacuation time of exits 5, 6, 11, and 12 near the wave trough has increased. The distribution of evacuation time of each exit is more balanced, and the final evacuation time of each exit is closer.

**Figure 7:**
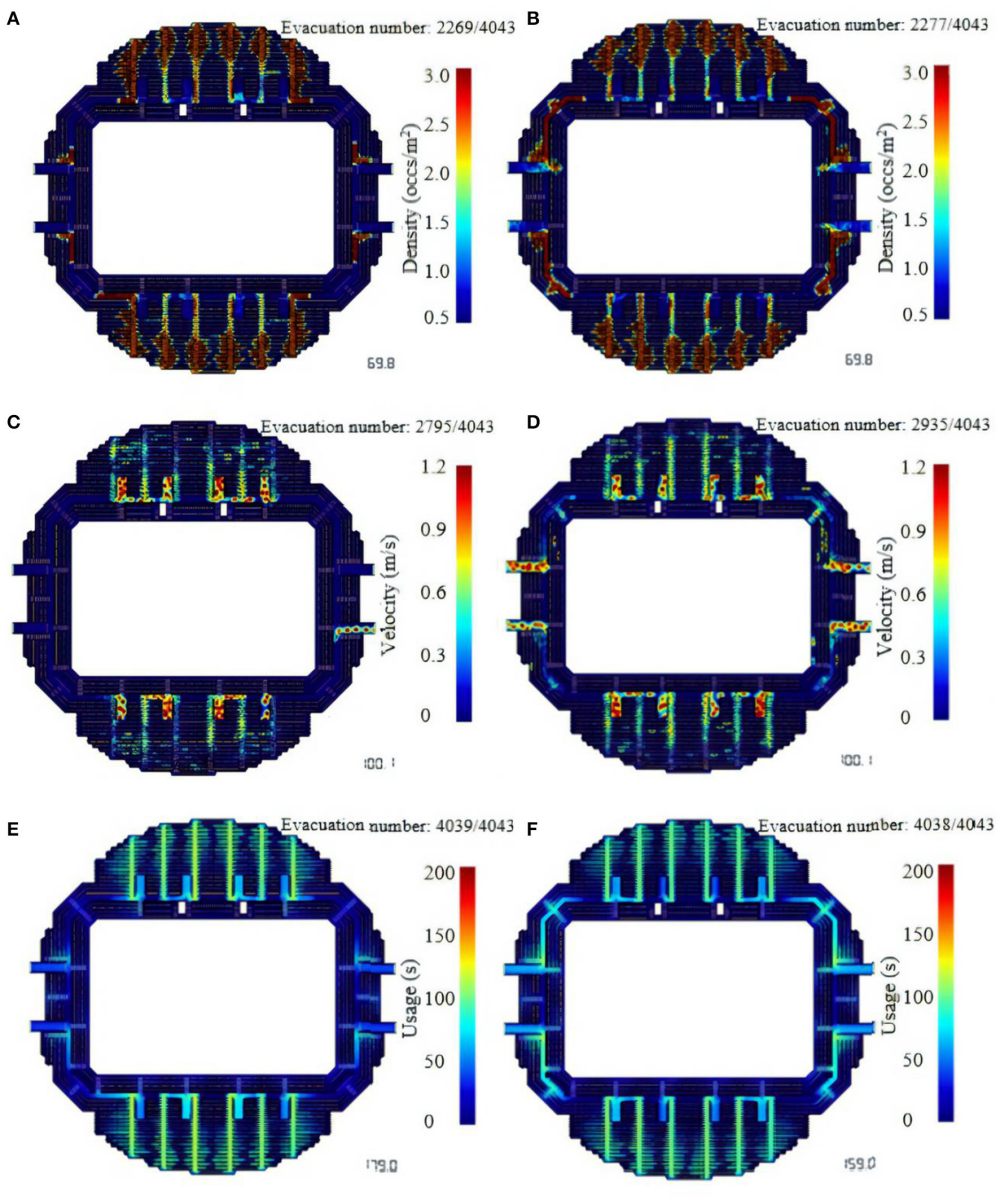
Evacuation status at a certain moment in the evacuation scene, (left: before optimization, right: after optimization).

Figure 7 shows the density and speed distribution of evacuees at a certain time in the stadium before and after the optimization, and the distribution of the last use time. Before evacuation, due to the shortest path evacuation principle, people will flock to the nearest exit. Before optimization, congestion mainly occurred in the seating area and the descending stairs leading to exits 1, 2, 3, 4, 7, 8, 9, and 10. Small-scale congestion occurred in the corridors near exits 5, 6, 11, and 12, as shown in the red area in Figure 7A; after the model was optimized for partitions, the congestion at the seats and stairs was relieved, and the congestion pressure was distributed to the corridor part of the path leading to exits 5, 6, 11, and 12. It can be seen from Figures 7C,D that when the stadium was evacuated to 100.1 s before optimization, exits 5, 11, and 12 have been evacuated and are in an idle state. Exit 6 is about to complete evacuation while the remaining exits are still in severe congestion, some people cannot reach the stairs from the seating area and are almost at a standstill (0-0.12 m/s). After optimization, the vicinity of each exit is in a balanced utilization state, and most of the people who use exits 1, 2, 3, 4, 7, 8, 9, and 10 as evacuation exits have left the seating area and converged to the stairs to queue for evacuation. In Figures 7C,D, the use time of the north-south evacuation passage is within 100~120 s, the use time of the east-west evacuation passage is generally <60 s, and only a small part is in the 80-100 s. After optimization, it is obvious that the east-west evacuation passages share the flow of people on the north-south passage, and the evacuation efficiency has been significantly improved.

### Vertical Evacuation

#### Optimization Strategy

For most high-rise hospitals, the layout of departments is not planned from the perspective of safe evacuation. Therefore, some departments with a high proportion of severely disabled patients will be arranged on the higher floors of the hospital. In the event of a fire, these patients have poor mobility and a large vertical distance from the ground. It takes a long time to evacuate to a safe area. Moreover, these patients usually need other people or equipment to assist in the evacuation, which will cause more serious congestion in the stairway, so it will greatly reduce the overall evacuation efficiency of the hospital.

In multi-floor public places, due to the differences in the functions of each floor in the vertical space, there are also differences in the composition and proportion of personnel. Therefore, whether the floor layout optimization is scientific and reasonable will affect the utilization efficiency and congestion status of the vertical evacuation passages on the floor to a certain extent, thereby affecting the overall safety evacuation efficiency of the building.

In the high-rise hospital scene in this article, according to different floor distribution, the departments mainly include Inpatient Department, Dermatology Department, Respiratory Department, Neurology Department, E. N. T. Department, Endocrinology Department, Neurosurgery Department, Cardiovascular Department, Plastic surgery, Oncology Department, Surgery Department, Pediatric Department, Orthopedics Department, Gynaecology and obstetrics, Cardiothoracic surgery and ICU (Intensive Care Unit). Based on the on-site statistical data of the hospital, this paper analyzes the number and proportion of personnel in different departments and categories and divides the evacuation levels of each floor into levels I, II, and III according to their evacuation capabilities, as shown in Table 5.

**Table 5:** Evacuation level of each department.

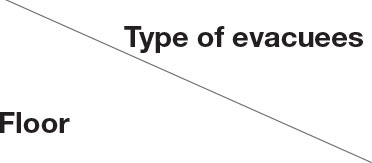	**Normal person**	**Mild disabled person**	**Severe disabled person**	**Complete disabled person**	**Total**	**Evacuation level**
**Floor**						
Impatient Department	131	7	11	3	150	/
Dermatology Department	121	12	1	1	135	I
Respiratory Department	65	18	4	1	89	I
Neurology Department	57	22	5	2	87	I
E. N. T. Department	77	26	5	3	111	I
Endocrinology Department	85	33	6	3	130	I
Neurosurgery Department	47	52	8	6	112	II
Cardiovascular Department	38	56	6	5	107	II
Plastic Surgery	40	36	9	7	91	II
Oncology Department	66	32	14	6	118	II
Surgery Department	51	15	27	8	101	II
Pediatric Department	76	14	31	2	123	III
Orthopedics Department	28	36	25	8	97	III
Gynaecology and Obstetrics	55	26	19	17	117	III
Cardiothoracic Surgery	49	35	16	21	121	III
ICU	29	34	13	28	104	III

**Figure 8:**
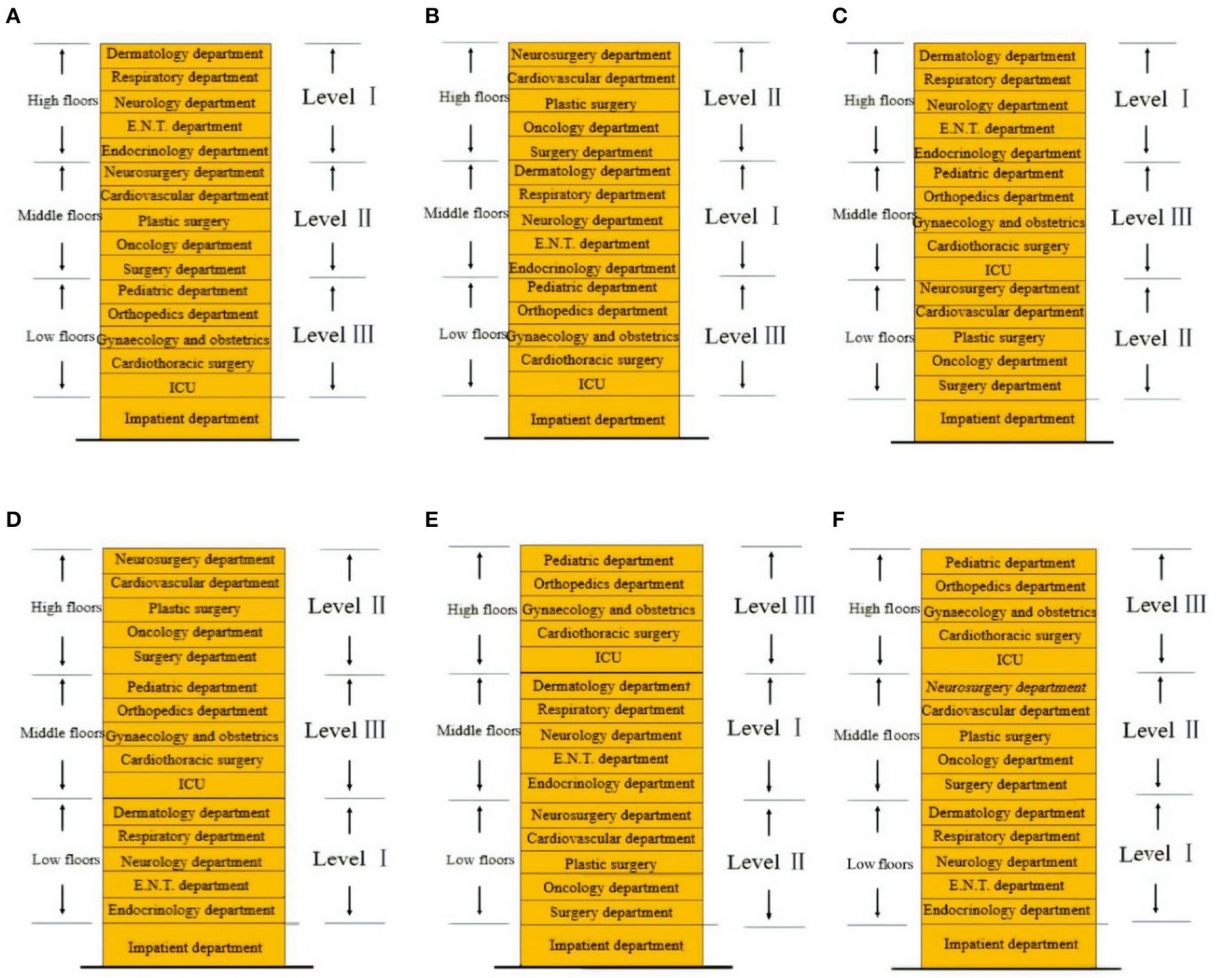
High-rise hospital layouts. **(A)** Layout 1. **(B)** Layout 2. **(C)** Layout 3. **(D)** Layout 4. **(E)** Layout 5. **(F)** Layout 6.

In the above, each floor is divided into Type I, II, and III levels according to the evacuation capacity. According to the height of the floor, a total of 15 floors from the second to the sixteenth floors can be divided into high, middle, and low floors. In order to study the relationship between the vertical layout of the departments and the efficiency of safe evacuation, this paper arranges the departments of the I, II, and III evacuation levels with high, middle, and low floor heights, and a total of 6 layout modes, as shown in Figure 8. Through the evacuation simulation analysis of these six layout modes, the optimal evacuation optimization strategy is explored.

#### Results

**Figure 9:**
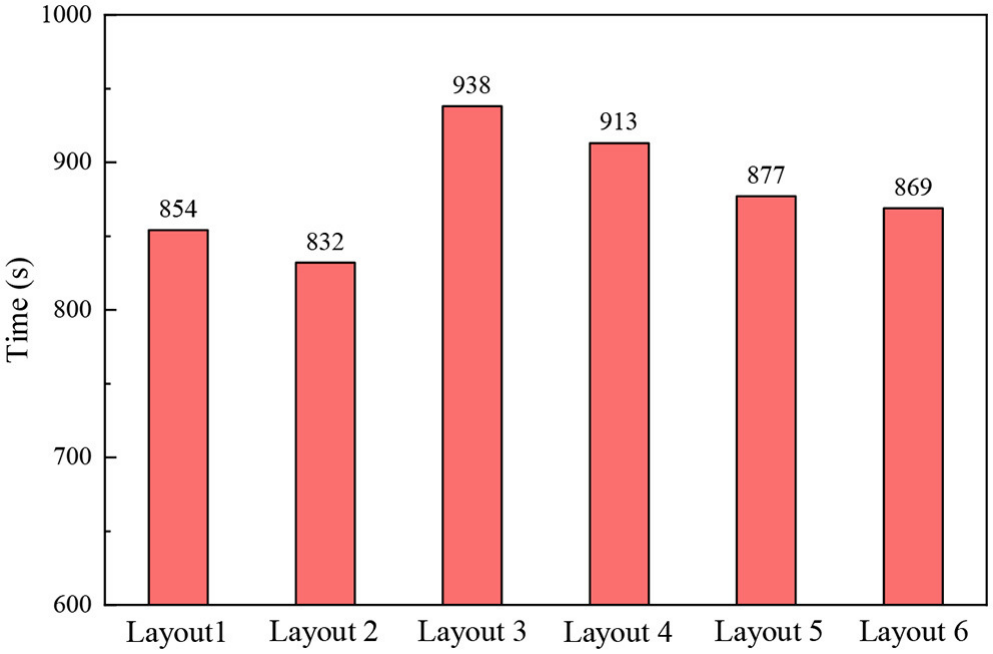
Evacuation time under different layouts.

From Figure 9, we can see the results of the evacuation simulation, the shortest overall evacuation time is layout 2, which only took 832 s. In this layout mode, the level III departments with the worst evacuation capabilities are placed in the low floor, the level I departments with the best evacuation capabilities are placed in the middle floor, and the level II departments with medium mobility capabilities are placed in the high floor. The evacuation time closest to layout 2 is layout 1, and the overall evacuation time is 854 s. Through comparison, it can be found that the two have the same point in that they both arrange level III departments on the floor closest to the ground in the hospital. Layouts 3 and 4 have the worst evacuation efficiency and the longest evacuation time, 938 and 913 s, respectively. Both of these layouts place level III departments on the high floor that are the furthest vertical distance from the ground. The evacuation efficiency of layouts 5 and 6 is at a medium level, and the evacuation time is 877 and 869 s, respectively.

The evacuation process shows that when the level III departments are arranged closest to the ground, the following effects will be caused: (1) In the early stage of evacuation, the evacuation speed of the lower floors of the hospital is slower than that of the middle and high floors. Because the lower floors are connected to the outside world through safe evacuation exits, the overall evacuation efficiency of the hospital building is also slower in the initial stage. However, since most of the people on each floor are still in the plane evacuation stage at this time and have not moved to the stairs, this strategy will not have a great impact on the evacuation speed of the high floors. (2) In the middle stage of evacuation, evacuees from level III departments with poor athletic ability are very close to the ground, so they can quickly evacuate with the help of medical staff. When the people from the middle and high floors are evacuated to the low area, most of the evacuees in the low area have been evacuated, and there will be no congestion.

In the departments of III evacuation level, the proportion of patients evacuated by wheelchairs and beds is higher, which will occupy more space and be more prone to congestion. Arranging evacuees with better evacuation ability on the middle floor can make the evacuation congestion area closer to the ground and reduce the evacuation efficiency drop caused by congestion. Therefore, placing the evacuees with the worst evacuation ability on the lowest floor, the evacuees with the best evacuation ability on the middle floor, and the evacuees with middle evacuation ability on the highest floor can effectively optimize the overall evacuation efficiency of high-rise hospitals.

## Conclusions

This paper mainly studies the evacuation optimization strategy for two types of public places divided according to the spatial characteristics of the building and establishes the evacuation models of large stadiums and high-rise hospitals, respectively.

Aiming at the evacuation problem of large stadium, with the shortest overall evacuation time as the optimization purpose, an optimization method for evacuation zones is proposed. The study pointed out that the strategy of partition evacuation can effectively improve the evacuation efficiency of the stadium, reducing the total evacuation time by 10.55%. Partition evacuation optimizes the initial exit choice of the evacuees so that the evacuation demand of each exit is more balanced, which can effectively avoid the crowding phenomenon at the exit. In the study of evacuation strategies for high-rise hospitals, the number of evacuees and physical conditions on different floors were compared and sorted, and the departments were divided into evacuation levels of I, II, and III. The departments of I, II, and III evacuation levels are arranged and combined with high, middle, and low floors to produce a total of 6 layouts. Numerical simulation results indicate that the evacuees with the worst evacuation capacity are placed on the lowest floor, the evacuees with the best evacuation capacity are placed on the middle floor, and the evacuees with medium evacuation capacity are placed on the highest floor with the highest evacuation efficiency.

This paper uses model simulation methods to study the horizontal and vertical evacuation of public places. According to different evacuation characteristics, corresponding evacuation optimization strategies are proposed, which proves the effectiveness of the evacuation strategy and can provide suggestions for the layout and planning of large public places.

## Data Availability Statement

The original contributions presented in the study are included in the article/supplementary material, further inquiries can be directed to the corresponding author/s.

## Author Contributions

HF: funding acquisition, validation, conceptualization, and writing—original draft. HF and WL: formal analysis, methodology, and writing—review and editing. HC and ZS: writing—review and editing, funding acquisition, validation, and supervision. XL and BY: validation and writing—review and editing. All authors contributed to the article and approved the submitted version.

## Funding

This study was supported by grants from the National Natural Science Foundation of China (Nos. 51978573 and 51778529).

## Conflict of Interest

The authors declare that the research was conducted in the absence of any commercial or financial relationships that could be construed as a potential conflict of interest.

## Publisher's Note

All claims expressed in this article are solely those of the authors and do not necessarily represent those of their affiliated organizations, or those of the publisher, the editors and the reviewers. Any product that may be evaluated in this article, or claim that may be made by its manufacturer, is not guaranteed or endorsed by the publisher.
